# Assessment of MRI image distortion based on 6 consecutive years of annual QAs and measurements on 14 MRI scanners used for radiation therapy

**DOI:** 10.1002/acm2.13843

**Published:** 2022-11-16

**Authors:** Lanchun Lu, Xiangyu Yang, Brian Raterman, Xia Jiang, Matthew Meineke, John Grecula, Dukagjin Blakaj, Joshua Palmer, Raju Raval, Evan Thomas, David Hintenlang, Nilendu Gupta

**Affiliations:** ^1^ Department of Radiation Oncology The Ohio State University Columbus Ohio USA; ^2^ Department of Radiology The Ohio State University Columbus Ohio USA

**Keywords:** imaging isocenter, margin, MRI image distortion, MRI image phantoms, region of interest

## Abstract

**Purpose:**

To determine the magnitude of MRI image distortion based on 6 consecutive years of annual quality assurances/measurements on 14 MRI scanners used for radiation therapy and to provide evidence for the inclusion of additional margin for treatment planning.

**Methods and materials:**

We used commercial MRI image phantoms to quantitatively study the MRI image distortion over period of 6 years for up to 14 1.5 and 3 T MRI scanners that could potentially be used to provide MRI images for treatment planning. With the phantom images collected from 2016 to 2022, we investigated the MRI image distortion, the dependence of distortion on the distance from the imaging isocenter, and the possible causes of large distortion discovered.

**Results:**

MRI image distortion increases with the distance from the imaging isocenter. For a region of interest (ROI) with a radius of 100 mm centered at the isocenter, the mean magnitude of distortion for all MRI scanners is 0.44±0.18mm, and the maximum distortion varies from 0.52to1.31mm depending on MRI scanners. For an ROI with a radius of 200 mm centered at the isocenter, the mean magnitude of distortion increases to 0.84±0.45mm, and the range of the maximum distortion increases to 1.92−5.03mm depending on MRI scanners. The distortion could reach 2 mm at 150 mm from the isocenter.

**Conclusion:**

An additional margin to accommodate image distortion should be considered for treatment planning. Imaging with proper patient alignment to the isocenter is vital to reducing image distortion. We recommend performing image distortion checks annually and after major upgrade on MRI scanners.

## 1 INTRODUCTION

MRI images have been widely used for treatment planning of radiation therapy, where they are used for delineating tumor targets and organs at risk (OARs) during the treatment planning process. This utilizes MRI's unique capability of identifying targets and OARs in soft tissues compared with using CT images only. Additionally, the functional image feature of MRI has the potential capability for physicians to assess treatment outcomes through radiomics studies.^1^ Modern radiation therapy aims to deliver maximum dose to tumors while reducing the dose to OARs and healthy tissues. The route to achieve this goal is to reduce clinical target volumes and planning target volumes by reducing the margins that are expanded from gross tumor volumes. These margins accommodate the uncertainties from microscopic disease, interobserver variation during target delineation, organ motion, and patient setup. Unlike CT images, MRI images have a considerable magnitude of geometrical distortion due to the scanner‐related inhomogeneities of magnet fields and the signal processing–dependent artifacts of MRI scanners.^2^, ^3^, ^4^ Image distortion means that a position showed on an MRI image with the coordinate of (x,y,z) is indeed different from what it really locates. In other word, there is a deviation between what one sees on the MRI image and what is its real location, and the actual coordinate should be (x+Δx,y+Δy,z+Δz). The absolute magnitude of deviation $\left(\Delta x,\;\Delta y,\;\Delta z\right),\;\sqrt{{\left(\Delta x\right)}^{2}+{\left(\Delta y\right)}^{2}+{\left(\Delta z\right)}^{2}}$, could be up to several mms or even worse.^5^, ^6^, ^7^, ^8^ This deviation leads to inaccurate delineations of targets and OARs as well as the margin determination in a treatment plan, and for some challenging scenarios utilizing small targets such as trigeminal neuralgia, tremors, obsessive compulsive disorder (OCD) in Gamma Knife radiosurgery or other SBRT/SRS treatments where a high dose is being delivered; this deviation may result in missing the treatment target or causing unexcepted damage to OARs. Although the issue of MRI image distortion is well known and has been qualitatively studied, the detailed quantitative investigation has not been well emphasized and researched until recently.^9^, ^10^, ^11^, ^12^ In this paper, we present our quantitative studies across a 6‐year period on the image distortion of 14 MRI scanners (1.5 and 3 T of GE and Siemens scanners) that could potentially be used to provide MRI images for treatment planning of radiation therapy. The results from this study may provide a reference to the field of radiation therapy of whether an additional margin attributed from MRI distortion needs to be included when MRI images are used for treatment planning and treatments.

## 2 METHODS AND MATERIALS

We used commercial MRI phantoms called MRID^3D^ and GRID^3D^ (Modus QA, Ontario Canada N6H 5L6, https://modusqa.com/) to acquire images on MRI scanners and perform the annually image distortion quality assurance (QA) checks. MRID^3D^ is a cylinder‐like phantom with the physical dimensions of 39.4 cm (diameter) ×39.4cm (length). The phantom is filled with paraffinic mineral oil and contains a certain number of coordinate‐known control points that are used as references to calculate the deviations due to the image distortion. The image dimension of the phantom is 36.8 cm (diameter) ×32.1cm (length). GRID^3D^ phantom is a much smaller image distortion QA phantom that was originally designed for Gamma Knife imaging QA check. To measure image distortion, GRID^3D^ phantom must attach with a Gamma Knife head frame and the fiducial box for imaging. The illustration of (a) MRID^3D^ phantom and (b) GRID^3D^ phantom can be found on the Supporting Information File#1. We scanned these phantoms on 14 1.5 and 3 T MRI scanners that provided MRI images for radiation therapy treatment planning. Scans were performed annually or whenever there was a major upgrade either on hardware or software since 2016 when we first used these phantoms to quantitatively measure the MRI image distortion and monitored the stability of image distortion over the period of 6 years. The phantoms were scanned on these MRI scanners with 1 mm image slice thickness and a T1‐weighted 3D sequence by following the imaging protocol of Gamma Knife radiosurgery and Linac‐based SBRT/SRS as shown in Table 1. For each imaging scan, the phantom was aligned to its geometric center using the MRI's lasers. The field‐of‐view was set to cover the entire phantom. The acquired images were exported to the computer with the MRID^3D^ or GRID^3D^ image analysis software that was specifically designed by the manufacturer Modus QA for the MRID^3D^ phantom and GRID^3D^ phantom to measure the image distortion of MRI scanners. Phantom images collected from 2016 to 2022 were analyzed to evaluate the distortion for various of regions of interest (ROIs), defined as a sphere with the origin at the imaging isocenter (0,0,0) with various radii in the unit of mm: (1) the absolute magnitude of the image distortion and the distortions on *x*‐, *y*‐, and *z*‐axis; (2) the dependence of distortion magnitude on the distance from the imaging isocenter; (3) the possible difference in the magnitude of image distortion between 1.5 and 3 T scanners; (4) the trend and the stability of image distortion for each MRI scanner across the past 6 years; (5) the possible causes of large image distortion for some scanners, and (6) the general magnitude of image distortion for MRI scanners that may provide the information for the additional treatment margins needed to accommodate MRI image distortion for the delineation of targets and OARs in radiation therapy.

**TABLE 1:** MRI scan protocols for the small phantom GRID^3D^ and the large phantom MRID^3D^

**Small phantom (GRID^3D^) protocol**			**Large phantom (MRID^3D^) protocol**		
**Scanner**	**1.5 T**	**3 T**	**Scanner**	**1.5 T**	**3 T**
**Coil**	T/R head	N/A—the small phantom was not scanned at 3 T	**Coil**	T/R body	T/R body
**Pulse sequence**	3D FLASH/SPGR		**Pulse sequence**	3D FLASH/SPGR	3D FLASH/SPGR
**Field‐of‐view (FOV, mm)**	250 x 250 or 256 x 256		**Field‐of‐view (FOV, mm)**	448 x 448	448 x 448
**Pixel size (mm)**	0.8 x 0.8 or 1 x 1		**Pixel size (mm)**	1 x 1	1 x 1
**Slice thickness (mm)**	0.8 or 1		**Slice thickness (mm)**	1	1
**Repetition time (TR, ms)**	Min (7.8–9.0)		**Repetition time (TR, ms)**	Min (7.8–9.6)	Min (6.0–6.5)
**Echo time (TE, ms)**	Inphase (∼4.4)		**Echo time (TE, ms)**	Inphase (∼4.4)	Inphase (∼2.2)
**Flip angle (FA, degree)**	10.5–20.0		**Flip angle (FA, degree)**	10.5–25	10.5
**Bandwidth (Hz/Pixel)**	130–150		**Bandwidth (Hz/Pixel)**	150–279	230
**Distortion correction**	3D		**Distortion correction**	3D	3D
**Time of acquisition (min)**	5:30–8:15		**Time of acquisition (min)**	20:30–28:11	16:52–20:06

## 3 RESULTS

### 3.1 MRI image distortion and the comparison of all 14 MRI scanners

We performed quantified image distortion annual QA checks using MRID^3D^ phantom and GRID^3D^ phantom on 14 MRI scanners at our medical center as early as in 2016. Among these 14 scanners, 6 were 1.5 T, and 7 were 3 T scanners, most of them were manufactured by Siemens Healthineers and one by GE Healthcare 1.5T. From the image distortion QA checks, only one scanner was found to have unexpectedly large image distortion and was recommended not to be used for radiation therapy treatment planning. The remaining 13 scanners demonstrated similar levels of image distortion. Table 2 presents the measured image distortion of these 13 scanners: Table 2A for the ROI with the radius of 200 mm; Table 2B for the ROI with the radius of 100 mm. For the ROI with the radius of 200 mm the mean magnitude of image distortion is 0.84±0.45mm, the maximum distortion varies from 1.92 to 5.02mm for different scanners as shown in Table 2A. If the radius of ROI is reduced to 100 mm, which is about the size of normal adult human being's head, the mean magnitude of image distortion decreases to 0.44±0.18mm, and the maximum distortion varies from 0.52 to 1.31mm for different scanners, as shown in Table 2B. These results indicate that for Gamma Knife radiosurgery, each of these 13 MRI scanners meets the criteria of 1 mm tolerance of image distortion. For other body parts where the tumors may be more than 100 mm from the imaging isocenter, the distortion is likely larger than 1 mm, an additional margin attributable to the MRI image distortion may need to be considered for treatment planning. However, the maximum distortion ranging from 0.52to1.31mm for different MRI scanners indicates that for some MRI scanners even when an ROI is within 100 mm away from the MRI imaging isocenter the magnitude of image distortion may still be larger than 1 mm, although it is not too much larger than 1 mm. Similarly, for an ROI with the radius of 200 mm where the distances of measurement/control points away from the MRI imaging isocenter are within 200 mm, although the mean magnitude of image distortion of all 13 MRI scanners is still under 1 mm (but increases to 0.84±0.45mm compared with the ROI with the radius of 100 mm), the maximum distortion ranging from 1.92to5.03mm indicates that for some MRI scanners even when an ROI is within 200 mm away from the MRI imaging isocenter the magnitude of image distortion may still be larger than 1.9 mm or even more to 5 mm.

**TABLE 2A:** Image distortion measured results: for the region of interest (ROI) with the radius of 200 mm for 13 MRI scanners

Dicom isocenter (0,0,0)	ROI: radius = 200 mm				
MRI scanners	Mean (mm)	STD (mm)	Max (mm)	*P*95 (mm)	% Above 2.50 mm
GE 1.5T Artist	0.555	0.309	2.171	1.162	0.000
Siemens 3T Prisma	0.626	0.340	2.318	1.318	0.000
Siemens 3T Verio1	1.034	0.509	5.028	1.952	2.090
Siemens 1.5T Sola1	0.694	0.317	1.933	1.270	0.000
Siemens 1.5T Sola2	0.496	0.249	1.920	0.945	0.000
Siemens 3T Verio2	1.015	0.625	4.786	2.199	3.030
Siemens 1.5T Aera1	0.681	0.339	3.103	1.307	0.019
Siemens 3T Skyra1	0.855	0.509	4.232	1.850	1.271
Siemens 1.5T Aera2	1.052	0.504	3.898	2.002	1.602
Siemens 3T Verio2	0.974	0.555	3.948	1.961	1.190
Siemens 1.5T Sola3	0.994	0.412	2.718	1.661	0.049
Siemens 3T Skyra2	1.163	0.576	4.261	2.215	1.650
Siemens 3T Skyra3	0.830	0.597	4.518	2.092	2.474
Average of total 13 MRI scanners	0.84	0.45	Range: 1.92–5.03		

**TABLE 2B:** Image distortion measured results: for the region of interest (ROI) with the radius of 100 mm for 13 MRI scanners

Dicom isocenter (0,0,0)	ROI: radius = 100 mm				
MRI scanners	Mean (mm)	STD (mm)	Max (mm)	*P*95 (mm)	% Above 2.50 mm
GE 1.5T Artist	0.325	0.130	0.688	0.538	0.000
Siemens 3T Prisma	0.331	0.076	0.563	0.466	0.000
Siemens 3T Verio1	0.649	0.222	1.307	0.988	0.000
Siemens 1.5T Sola1	0.368	0.150	0.809	0.650	0.000
Siemens 1.5T Sola2	0.286	0.114	0.524	0.471	0.000
Siemens 3T Verio2	0.479	0.244	1.441	0.948	0.000
Siemens 1.5T Aera1	0.313	0.128	0.619	0.518	0.000
Siemens 3T Skyra1	0.416	0.193	1.219	0.785	0.000
Siemens 1.5T Aera2	0.547	0.198	1.073	0.900	0.000
Siemens 3T Verio2	0.465	0.215	1.026	0.846	0.000
Siemens 1.5T Sola3	0.511	0.216	0.978	0.864	0.000
Siemens 3T Skyra2	0.614	0.235	1.236	1.005	0.000
Siemens 3T Skyra3	0.372	0.196	1.147	0.724	0.000
Average of total 13 MRI scanners	0.44	0.18	Range: 0.52–1.31		

**FIGURE 1:**
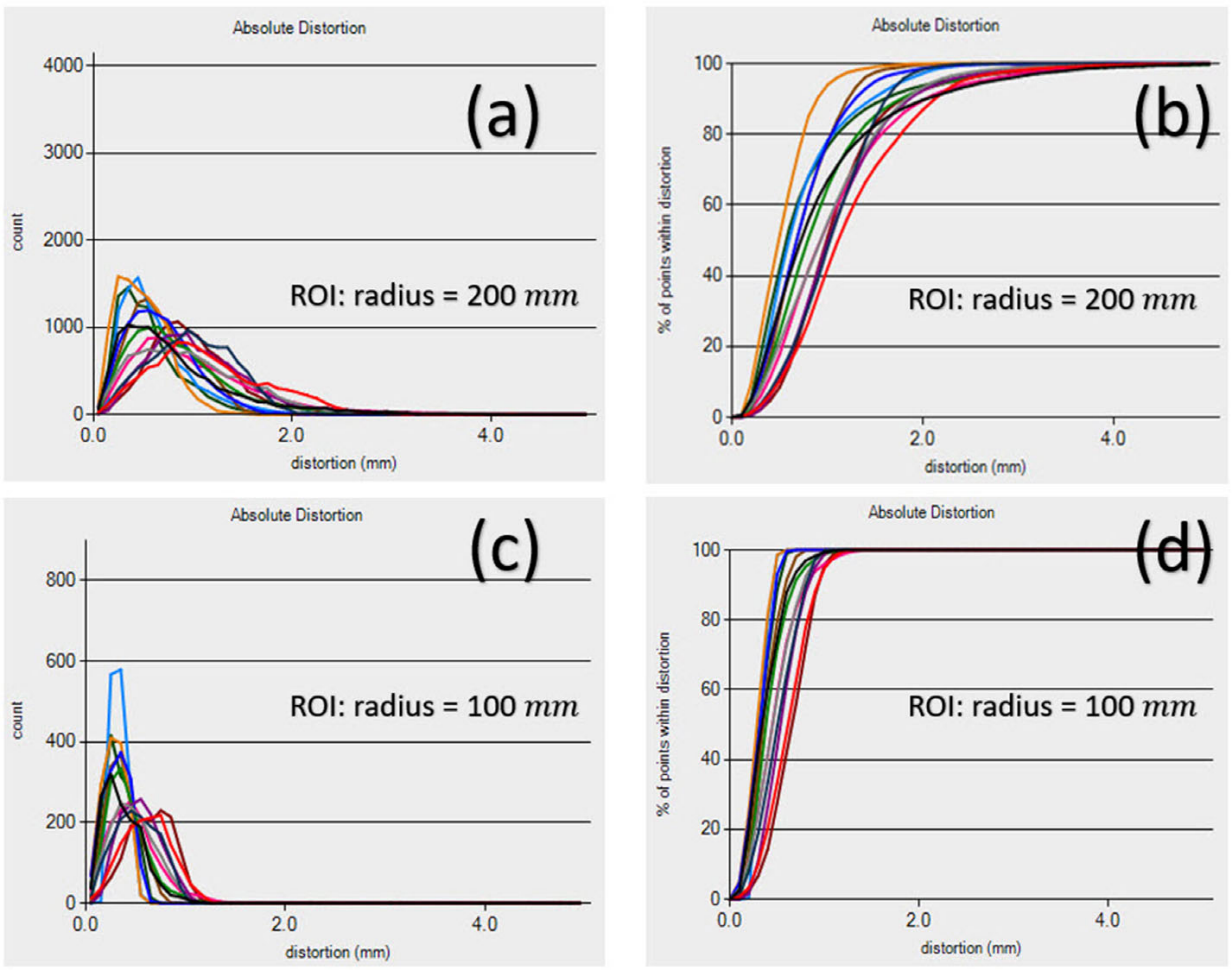
(a) The histogram (b) percentage distribution of control points versus image distortion absolute magnitude for the region of interest (ROI) within the radius of 200 mm for 13 MRI scanners, parts (c) and (d) are those for the ROI within the radius of 100 mm for 13 MRI scanners.

Figure 1 shows (a) the histogram and (b) percentage of control points versus image distortion absolute magnitude for the ROI with the radius of 200 mm, respectively, for 13 MRI scanners, whereas (c) and (d) are those for the ROI with the radius of 100 mm . From Figure 1, one can see for almost all 13 MRI scanners, when the ROI is within the radius of 100 mm, the distortion is predominantly less than 1 mm. For some scanners, there is a small percentage of points having the distortion slightly larger than 1 mm but much less than 2 mm. Although when the ROI extends to the radius of 200 mm, there are up to about 50% control points being distorted more than 1 mm and for some control points the distortion could reach to 2 mm or even more varying with individual scanner.

We studied the mean image distortion versus bandwidth for the ROI with the radius of 200 and 100 mm for these 13 MRI scanners. The effect of scanner hardware/software on the image distortion was found to be substantially larger than that of bandwidth, which can be seen in the Supporting Information File#2: (a) for the ROI with the radius of 200 mm and (b) for the ROI with the radius of 100 mm. In the distortion versus bandwidth plot on Supporting Information File#2, there are two bands of data points based on the bandwidth value on *X*‐axis. The higher bandwidth group is corresponding to the 3 T scanners, and the lower bandwidth group is for 1.5 T scanners. Bandwidth plays a big role in 3 T scanners. Small bandwidth will have larger distortion, thus on Supporting Information File#2 we can see that 3 T scanners used higher bandwidth to get a reasonable and comparable small distortion. The bandwidth we used for our MRI scanners can be found in our MRI protocol (see Table 1).

**FIGURE 2:**
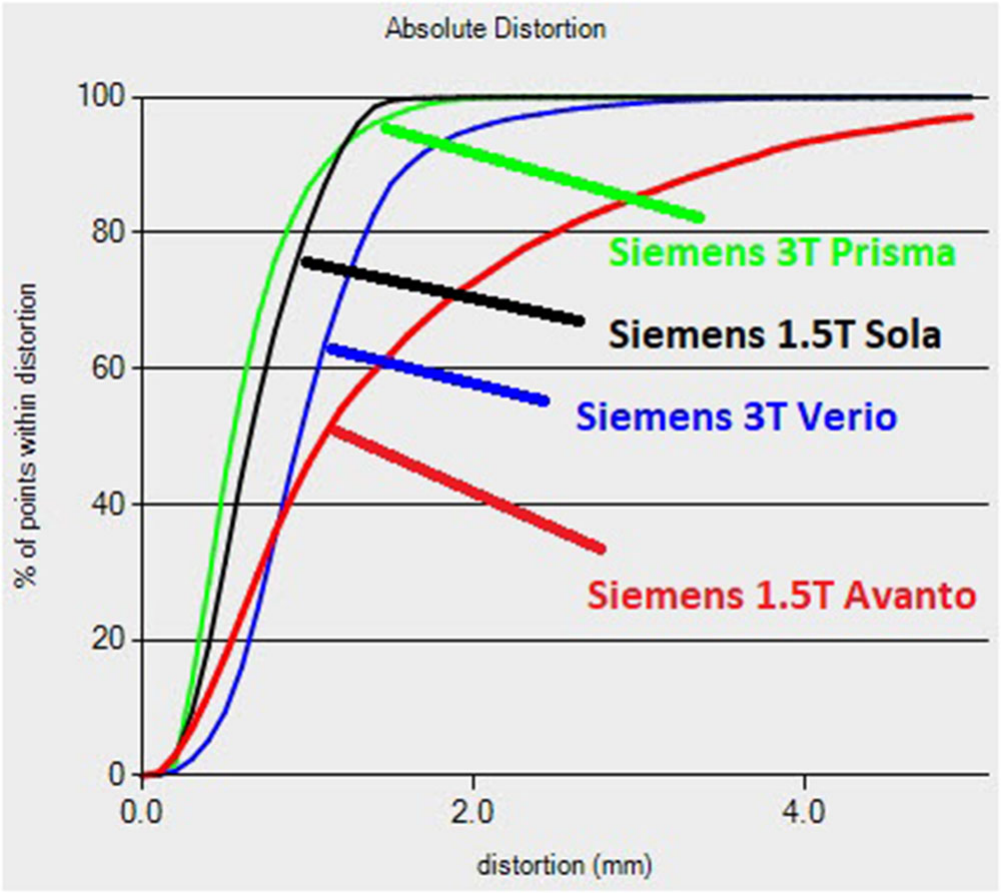
The problematic MRI machine Siemens 1.5T Avanto with large image distortion and the comparison with other scanners

As mentioned before among those 14 MRI scanners that we performed the quantified distortion QA checks, one of the oldest MRI scanners, Siemens 1.5T Avanto, was found to have an unacceptably large image distortion. We excluded this scanner from being used for radiation therapy. The mean magnitude of image distortion of this scanner is 1.61±1.54mm, with maximum magnitude of distortion up to 23 mm and with about 50% of control points being distorted ≥1mm and about 30% of control points being distorted ≥2mm. Figure 2 is the comparison of the image distortion for this MRI scanner with those for another Siemens 1.5T Sola and the other two Siemens 3T scanners, from which one can see the Siemens 1.5T Avanto has obvious larger image distortion compared with other three scanners as shown in Figure 2. The large distortion is likely due to the machine being too old to have the software support of 3D distortion correction.

### 3.2 Magnitude of MRI image distortion versus the distance away from the imaging isocenter

**FIGURE 3:**
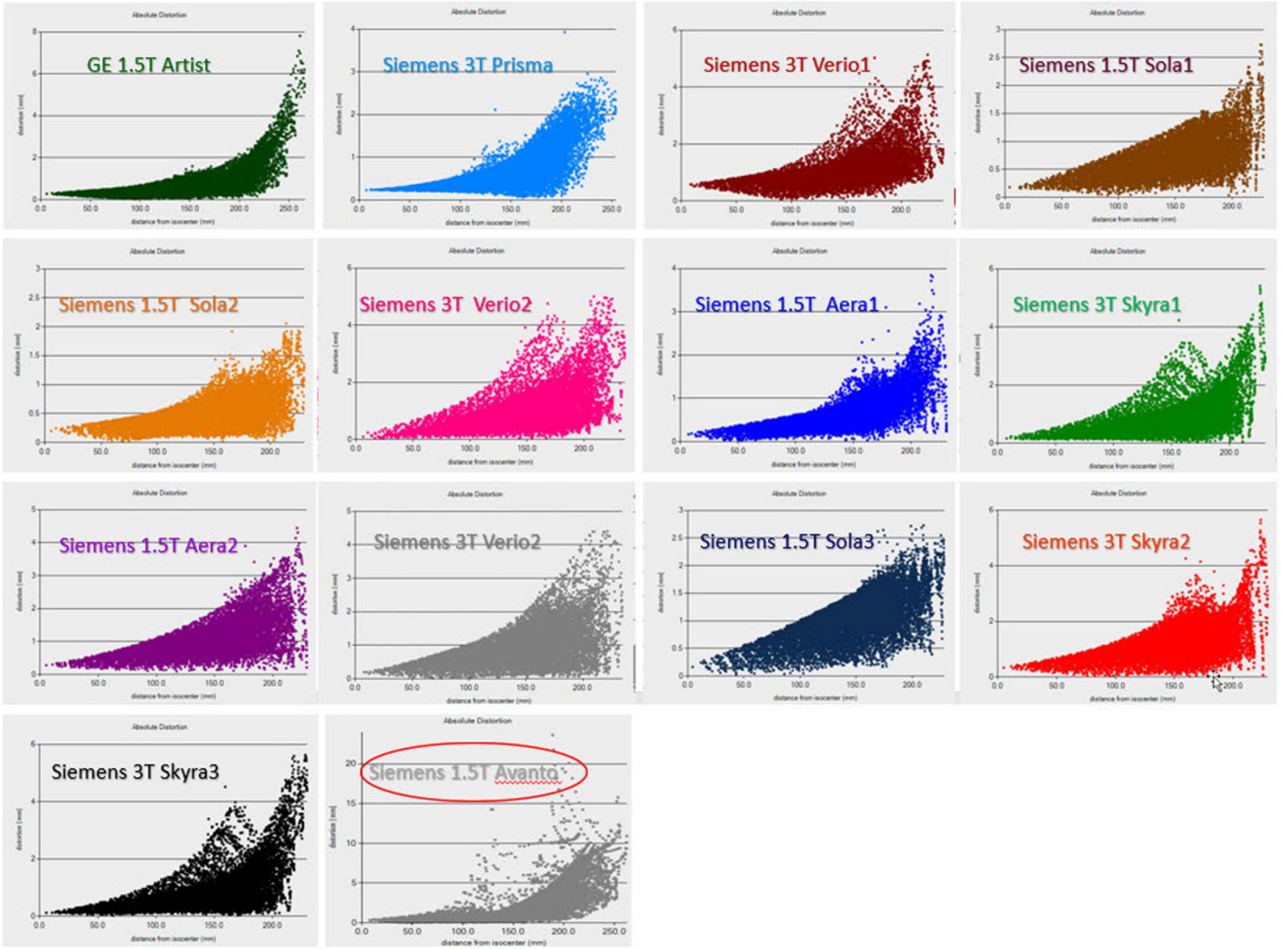
The magnitude of MRI image distortion versus the distance from the imaging isocenter for all the 14 scanners studied

We found that the magnitude of image distortion is strongly dependent on the distance away from the imaging isocenter for each scanner. Figure 3 shows the measured results of the magnitude of image distortion versus the absolute distance of the control points away from the imaging isocenter for all the 14 scanners, respectively. For all these MRI scanners, one can see that the magnitude of image distortion increases with the increase of the distance from the imaging isocenter. For some of these scanners, the magnitude of distortion can easily reach 2 mm when the distance from imaging isocenter is 150 mm. When the distance is close to 200 mm away from the imaging center, all of the MRI scanners have some control points with image distortion ≥2mm. Once again, one can see the Siemens 1.5T Avanto (the last graph in Figure 3) has a much larger image distortion compared to the other 13 scanners.

### 3.3 Stability of MRI image distortion for MRI scanners over the period of 6 years

We have been using the commercial MRI image phantom MRID^3D^ to perform annual quantified image distortion QA checks and monitor the stability of the distortion since 2016 for all the 1.5 and 3 T MRI scanners that provided images for the treatment planning of radiation therapy at our medical center. This study provides us a unique opportunity to observe the stability of the MRI image distortion across several years of monitoring a scanner. The image distortion measured results from 2016 to 2021 for three MRI scanners: GE 1.5T Optima/Artist (upgraded from Optima to Artist in 2022), Siemens 1.5T Aera2, and Siemens 3T Skyra1 for the ROI with the radius of 200 and 100 mm, respectively, can be found in Supporting Information File#3. Over the 6‐year period from 2016 to 2021, the magnitudes of the MRI image distortion for these scanners were relatively stable and did not have much variation. This may owe to the well maintenance of the scanners by following the QA guidelines like the ACR protocol and the AAPM TG‐284 to perform the routine maintenance service and the regular QA checks as well as the product quality of a scanner. For the ROI with the radius of 200 mm, the image distortion for most all the control points is ≤2mm. Almost all the control points have less than 1 mm distortion when their distances from the imaging isocenter are less than 100 mm, which is indicated by the ROI with the radius of 100 mm in Supporting Information File#3. Although the level of image distortion for each individual scanner shown in our results is rather stable, there is still some variation across years for each scanner. For 3 T scanners, it seems that the variation is larger compared with 1.5 T scanners. The observed differences over time were likely due to spontaneous drift and periodic calibration of the gradient system.

### 3.4 Comparison of the magnitude of image distortion between 1.5 and 3 T scanners

We also compared the magnitude of image distortion between 1.5 T MRIs with 3 T MRIs, for both (a) ROI with the radius of 200 mm and (b) ROI with the radius of 100 mm. The detailed result of the comparison can be found in Supporting Information File#4. Overall 3 T scanners have slightly worse distortion compared with the 1.5 T scanners in terms of the mean magnitude of image distortion and the maximum range of distortion magnitude, which can also be seen in Figure 3. However, the difference on image distortion becomes less obvious when the distance to the imaging center is less than 100 mm as shown in Supporting Information File#4 (b) for the ROI with the radius of 100 mm. This could indicate that for brain patients where the imaging isocenter is likely near the center of patient's head, and the ROI is generally within the radius of 100 mm, using 3 T MRI scanners to acquire MRI images for Gamma Knife or Linac‐based SRS/SBRT treatment planning should be appropriate.

### 3.5 Comparison of the magnitude of image distortion between different vendors of MRI scanners

As we only included 14 MRI scanners from 2 vendors—1 GE MRI scanner and 13 Siemens scanners in this study, it is difficult to compare the image distortion between MRI scanners produced by different vendors. From the limited data we have analyzed, no significant difference on the image distortion for MRI scanners was found from different vendors. This could also be seen in Table 2 and Figure 3 as well as in the associated Supporting Information Files. It would be interesting to see the study results from other institutions if there are any similar studies.

### 3.6 Some examples that the magnitude of image distortion could be large and out of tolerance

During our 6 consecutive years of annual QAs using the MRID^3D^ phantom to measure the magnitude of image distortion on various MRI scanners used for radiation therapy, we occasionally found large image distortion for some MRI scanners from various causes. These findings remind us that the image distortion QA check for MRI scanners used for radiation therapy, especially for Gamma Knife, SBRT/SRS, or other types of radiosurgeries, is essential. The following are some examples that the image distortion was found being out of tolerance.

MRI scanner is too old, and the hardware/software cannot handle the image distortion to meet the criteria for Gamma Knife, SBRT/SRS, or other types of radiosurgeries. We used MRID^3D^ phantom to perform the image distortion check on an old Siemens 1.5T Avanto. Although this scanner was old, because patient volumes have increased and there is a lack of additional MRI scanners, it was proposed to use the scanner to scan patients for radiation therapy. Our measurement found that the mean magnitude of distortion was 2.25 ± 2.26 mm, with maximum distortion of 24 mm and 31% of control points having distortion larger than 2.5 mm as shown in Figures 2 and 3 for the scanner Siemens 1.5T Avanto.

**FIGURE 4:**
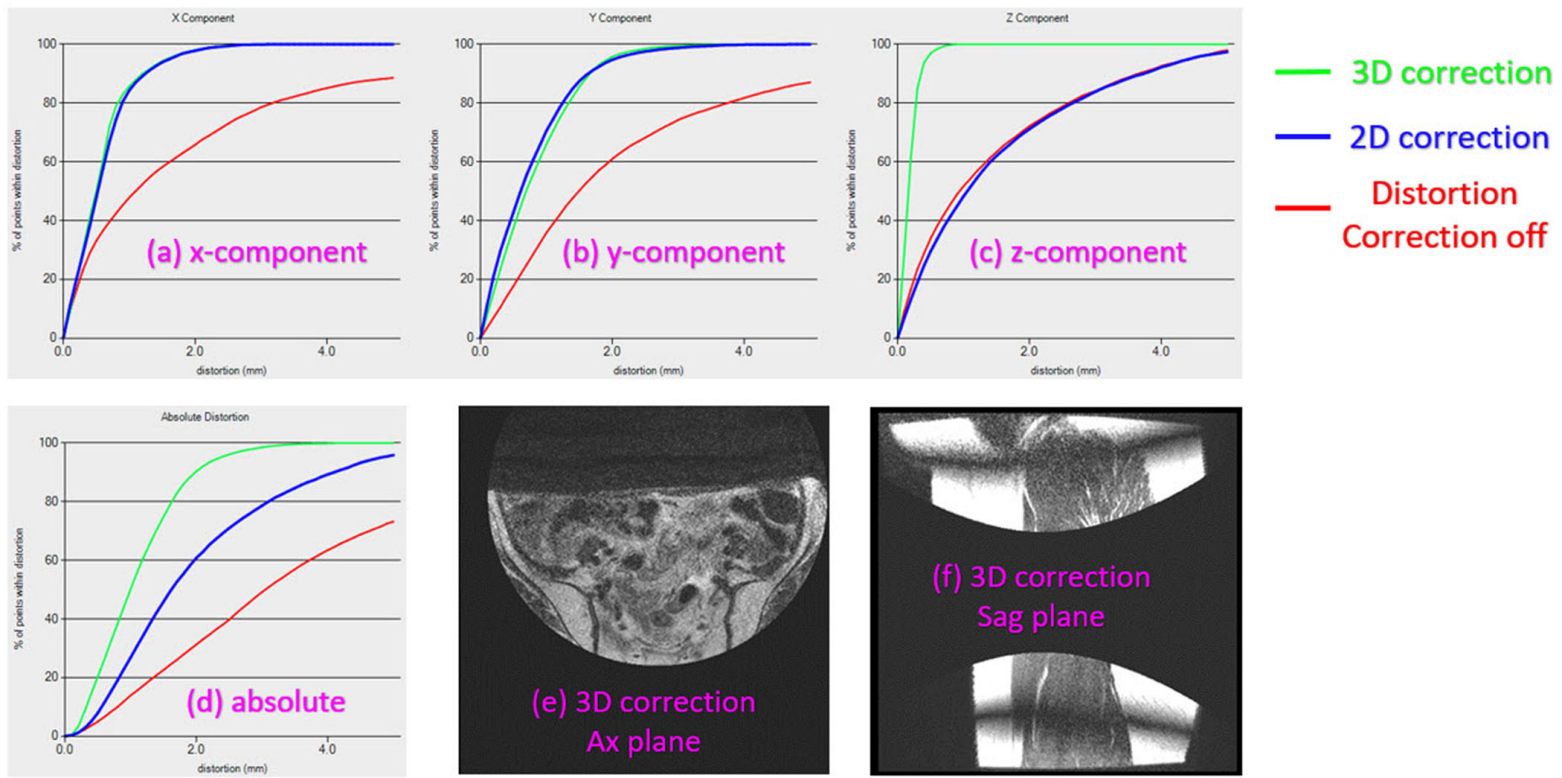
Comparison of the measured results of image distortion when 3D distortion correction was turned on, only 2D turned on, and distortion correction was completely turned off in respectively: (a)–(d) being the quantitative measurements of the image distortion on *x*‐, *y*‐, *z*‐direction, and the absolute magnitude in respectively for one of the Siemens 3T Verio scanner. Parts (e) and (f) are a patient's images on the axial (Ax) plane and the sagittal (Sag) plane, respectively.

MRI scanners have options on the console computers to turn on/off the 3D or 2D distortion correction. If the distortion correction was turned off accidently, the magnitude of image distortion was very large. 2D distortion correction only corrects the distortion in xy‐plane (only on *x*‐ and *y*‐direction, but not on *z*‐direction), whereas 3D correction corrects the distortions in all *x*‐, *y*‐, and *z*‐direction which will give more accurate results. Not only do all scanners have the option to turn on/off the distortion correction, is it OFF by default on both GE and Siemens, and the distortion correction must be enabled to take advantage of it. Siemens scanners specifically have the function of using 2D, or 3D distortion correction. GE scanners have 3D correction only for 3D scans as far as we were aware. If the 3D distortion correction is turned off accidently, the magnitude of image distortion will be very large even if the 2D distortion correction has been turned on—which only corrects the distortion on *x*‐ and *y*‐direction/component and leaves the *z*‐direction/component uncorrected. Figure 4 shows how the distortion corrections being turned on/off impact the magnitude of image distortion and the image quality. In the figure, we compared the measured results of image distortion for three scenarios: (1) 3D distortion correction was turned on, (2) only 2D correction was turned on, and (3) the distortion correction was completely turned off. Figure 4 from (a) to (d) are the quantitative measurements of the image distortion on *x*‐, *y*‐, *z*‐direction, and the absolute magnitude in respectively for one of the Siemens 3T Verio scanner. When 3D distortion correction was turned on, the magnitude of distortion is the smallest, whereas when distortion correction was completely turned off, the distortion is the largest. One of the ways to identify 3D distortion correction being used is to look at the image slices at the edge of the 3D slab. On axial images this will appear like a circular ring, as seen in Figure 4e,f, which is a patient's images on the axial plane and the sagittal plane, respectively. This is the result of shifting the pixel data in the slice direction. This appearance may be different depending on the slice orientation and position in the MRI scanner. However, this is usually quite difficult for a user who does not have much expertise to read MRI images. Figure 4 also indicates that merely by viewing a patient's MRI images while without performing a quantitative distortion QA check, it is not easy for a common MRI user to identify whether the image distortion correction has been turned on for an MRI scanner.

**FIGURE 5:**
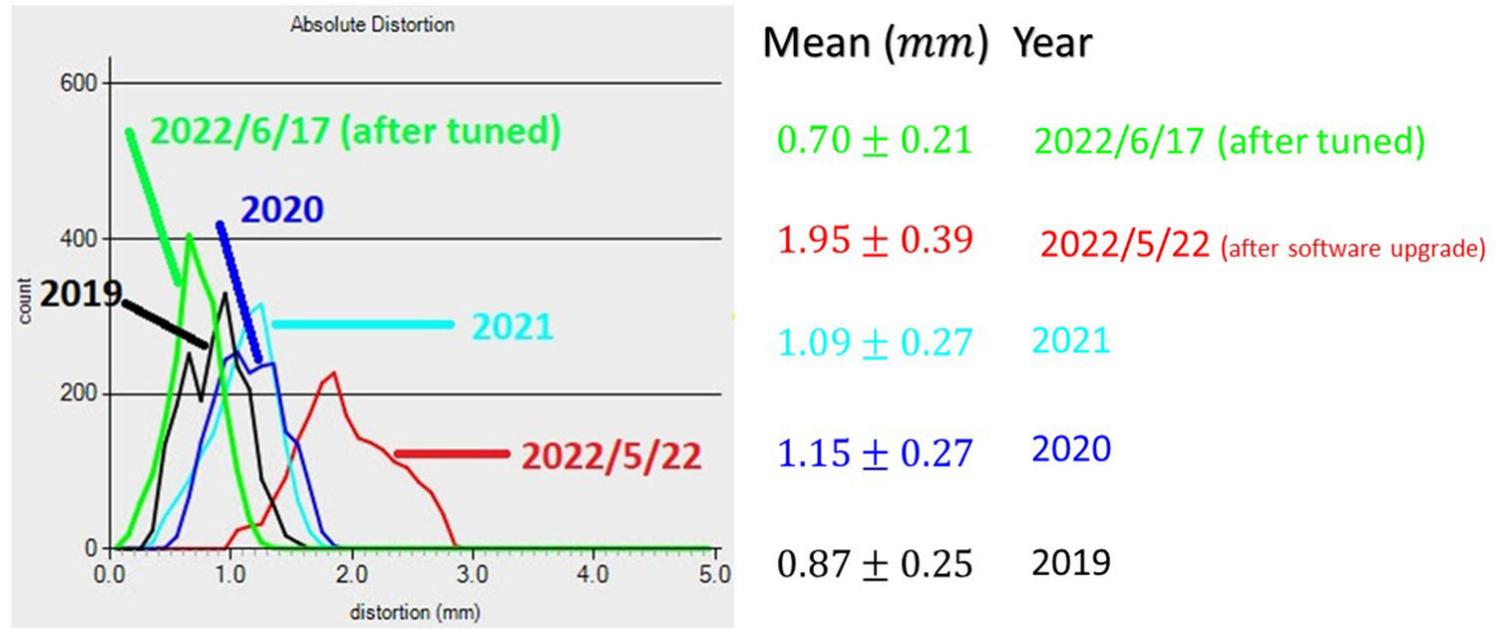
MRI image distortion after a major software upgrade (2022) and the comparison with previous measurements before the upgrade, 2021, 2020, 2019

After MRI scanners undergo a major software/hardware upgrade, the image distortion may get worse. If the major upgrade to the MRI hardware/software involves changes in the gradient system, shimming methods, or distortion correction algorithm, it may have noticeable impact on image distortion. Figure 5 shows the recent measured image distortion for an MRI scanner that had a major software upgrade recently (2022) and the comparison of the recent measurement with the measured results in the previous 3 years. The image distortion measurement was performed with the GRID^3D^ phantom with the Gamma Knife head frame and fiducial box attached, which was normally used to measure image distortion for frame‐based Gamma Knife patients. From the results shown in Figure 5, one can see after the software upgrade in 2022 (in red color dated 2022/5/22) that the mean magnitude of MRI image distortion is about 2 mm, which is much larger than the results measured in 2021, 2020, and 2019. This large distortion had been verified by co‐registration of the phantom's MRI image with its CT image and looking at the deviation of fiducial markers between the MRI and CT images. In general, CT images have almost no image distortion. For details one can see the Supporting Information File#5. The registration of the CT images with the MRI images after the software upgrade (2022) demonstrated about 3 mm deviation on the MRI fiducial markers (the left column in the figure), compared with almost no deviation between the CT images and the MRI images for the registration in 2021 (right column in the figure). Because the large distortion was identified, this MRI scanner was not utilized for imaging of Gamma Knife patients until the problem was fixed by the manufacture's service engineers. The result of the repeated distortion QA check after the engineer fixed the problem is also shown in Figure 5 (in green color, dated 17/6/2022). The verification of the correction can be found in Supporting Information File#6.

## 4 DISCUSSION AND CONCLUSIONS

We have quantitatively measured and monitored the MRI image distortion for 14 1.5 and 3 T MRI scanners used for radiation therapy over the period of 6 years. Our studies found that for radiation therapy or radiosurgery purposes the MRI image distortion cannot be neglected. For the position less than 100 mm away from the imaging isocenter, the image distortion can reach up to 1 mm and the magnitude of the distortion increases with the distance away from the imaging isocenter. At the position of 200 mm away from imaging isocenter, there is quite a large probability that the distortion could reach up to 2 mm or even larger. This indicates that when preparing MRI scans for radiosurgery or radiation therapy patients: (1) It is important to place the imaging isocenter at a proper position (such as at the center of ROI or as close as possible), which can reduce the image distortion for the ROIs, and (2) an additional planning margin resulting from MRI image distortion may need to be considered. The image distortion over 1 mm will have significant targeting and dose impacts for some procedures such as radiosurgery for trigeminal neuralgia or other functional brain diseases such as tremors, OCD, and pain release,^13^, ^14^, ^15^, ^16^ where extremely high doses are being delivered, and many critical OARs are near the targets.

Our study also shows that 3 T scanners have slightly larger magnitudes of image distortion compared with the 1.5 T scanners. For the position less than 100 mm away from the imaging isocenter, the difference of image distortion magnitude between 3 and 1.5 T is not significant if bandwidth is selected properly for a scanner. However, when the distance from the imaging isocenter increases the image distortion for 3 T scanners gradually gets worse compared with the 1.5 T scanners.

Our study reveals that over a period of 6 years, the magnitude of image distortion was quite stable, as long as an MRI scanner did not have major hardware or software upgrades, or if image distortion had been tuned and calibrated properly following an upgrade. There are many causes that may lead to large image distortion, such as age‐related scanner limitations, the image distortion option not being turned on in the console computers, or a scanner undergoing hardware or software upgrade, which indicates that regularly performing image distortion QA checks such as after a major hardware or software upgrade, or performing the QA annually, is very important. The differences on image distortion between scanners can be explained by the difference on the magnet of the individual scanner. Each scanner has a somewhat different magnet—even for the model from the same manufacturer, the magnet of a scanner may be slightly different. This difference determines the difference on homogeneity of the magnetic field that is used for acquiring signals for image reconstruction and hence leads to the difference on the image distortion. The quality of the image reconstruction software provided by different MRI manufactures may also play a role of the distortion difference between scanners made by different vendors. However, for the same scanner, most of the observed differences over time were likely due to spontaneous drift and periodic calibration of the gradient system. The drift across time could be due to the natural aging effect of RF coils or the magnet of the scanner that results in the change on the performance of the homogeneity of magnetic *B*0 field. This can explain why the distortion difference for a 3 T scanner is more sensitive than a 1.5 T scanner. Periodic calibrations or the calibrations due to a hardware/software upgrade or other reasons such as a problem discovered by routine annual or daily QAs, or a scanner being down due the failure of a part, etc., can also cause the difference on image distortion.

In some circumstances, an annual QA had already identified some quality index such as SNR of the scanner for example failed to comply the standard tolerance even before we performed a distortion QA check. Our quantitative image distortion QA check was usually performed as a part of the routine MRI annual QA but usually was scheduled at the end of the annual QA or after a major problem was discovered and solved. Although the current ACR guideline‐based distortion check recommended in the routine annual MRI QA could catch some cases of large image distortion as shown in the results, our experience tells us that under some circumstance ACR guideline‐based distortion check can miss some cases of large image distortion.

It is worth noting that MRI scanners may pass the ACR MRI accreditation protocol image distortion QA checks but may fail on our quantitative image distortion QA checks. This is because the distortion phantoms we used have many more control points and more detailed information. In addition, the criteria set for radiosurgery and radiation therapy are quite different from the one for diagnostic MRI images such as the ACR protocol for MRI image distortion QAs.

It should also be emphasized that the present study reports the measured image distortion inside uniform phantoms. The amount of distortion in patient images is expected to be larger due to inhomogeneities in patient anatomy and magnetic susceptibility, in which case the bandwidth and field strength may also have a larger impact on distortion than what was observed in this study. Further studies are needed to quantify the distortion in patient images.

We would like to point out that the results and conclusions reported in this paper are merely based on our experience on the 14 scanners from 2 vendors (13 Siemens and 1 GE scanners) that we have been monitoring and performing regarding quantitative image distortion QA checks, using the phantoms mentioned in the paper (MRID^3D^ and GRID^3D^) during the past several years. One certainly cannot expand all the conclusions universally to all scanners of different manufactures and modules. We hope to see other institutions in the near future that can report their study results on this similar topic so that the MRI and radiation therapy community can draw much broader generalization conclusions on MRI scanner performance. This will help the community improve the MRI imaging protocols and guidelines which essentially will enhance the quality of MRI images provided for radiation therapy.

We notice that the AAPM Task Group 284 had recently published the guideline for MRI imaging simulation in radiation therapy—Task group 284 report.^12^ This is an important milestone for the MRI and radiation therapy community, which arouses again the awareness of the importance of MRI imaging QA and the potential issues that may affect the quality of MRI images provided for radiation therapy and used for treatment planning, aside from the safety and other issues. It is a deep and broad guideline in which image distortion QA has been also included but mainly is based on the ACR protocol. Although the current TG 284 is an excellent and broad guideline for MRI simulation in radiation therapy, it would be better if it could recommend a much rigorous and detailed MRI distortion QA check aside from the current ACR protocol recommendations into the future updated version, as the current recommendation for the distortion check cannot provide a full picture of the detailed and quantified 3D distribution of the MRI image distortion, especially as the MRI distortion is a much more important issue in radiation therapy compared with in the world of diagnostic imaging, where the accuracy of coordinate for each image voxel is not as crucial as in the world of image‐guided radiation therapy, not to mention that higher accuracy is required for Gamma Knife radiosurgery and Linac‐based SRS/SBRT.

## AUTHOR CONTRIBUTIONS

All authors discussed the results and contributed to the manuscript. Lanchun Lu was responsible for statistical analysis.

## CONFLICT OF INTEREST

The authors declare that there is no conflict of interest that could be perceived as prejudicing the impartiality of the research reported.

## Supporting information

Supporting Information File#1 (a) The MRID^3D^ phantom, (b) GRID^3D^ phantom, (Modus QA, Ontario Canada N6H 5L6, https://modusqa.com/)
Supporting Information File#2 Show the mean image distortion magnitude versus bandwidth for the ROI with the radius of (a) 200 mm and (b) 100 mm for these 13 MRI scanners, respectively.
Supporting Information File#3 The stability of the magnitude of MRI image distortion over 6 years of period from 2021: (a) ROI with the radius of 200 mm; (b) ROI with the radius of 100 mm for GE 1.5T Artist, Siemens 1.5T Aera2, and Siemens 3T Skyra1
Supporting Information File#4 Comparison of the magnitude of image distortion between 1.5 and 3 T scanners: (a) ROI with the radius of 200 mm and (b) ROI with the radius of 100 mm
Supporting Information File#5 The registration of the CT images with the MRI images after the software upgrade (2022) showing about 3 mm deviation on the MRI fiducial markers (left column), compared with the registration of the CT and the MRI in 2021
Supporting Information File#6 The comparison of the registration of the CT images with the MRI images taken before and after the problem mentioned in Figure 5 and Supporting Information File#5 (due to the software upgrade in 2022) was fixed: (A) before the problem fixed showing about 3 mm deviation on the MRI fiducial markers (left column), (B) after the problem fixed showing the overlap of the MRI fiducial markers with the CT fiducial markers.

Supporting Information

Supporting Information

Supporting Information

Supporting Information

Supporting Information

Supporting Information

## Data Availability

All data generated and analyzed during this study are included in this published article (and its Supporting Information Files).
