# Supplementary material for: Assessment of MRI image distortion based on 6 consecutive years of annual QAs and measurements on 14 MRI scanners used for radiation therapy
Source: J Appl Clin Med Phys. 2022 Nov 16;24(1):e13843. doi: 10.1002/acm2.13843 (PMC9859981; doi:10.1002/acm2.13843)
Supplement: Supplementary file 1 — Supporting Information File#1 (a) The MRID3D phantom, (b) GRID3D phantom, (Modus QA, Ontario Canada N6H 5L6, https://modusqa.com/) Supporting Information File#2 Show the mean image distortion magnitude versus bandwidth for the ROI with the radius of (a) 200 mm and (b) 100 mm for these 13 MRI scanners, respectively. Supporting Information File#3 The stability of the magnitude of MRI image distortion over 6 years of period from 2021: (a) ROI with the radius of 200 mm; (b) ROI with the radius of 100 mm for GE 1.5T Artist, Siemens 1.5T Aera2, and Siemens 3T Skyra1 Supporting Information File#4 Comparison of the magnitude of image distortion between 1.5 and 3 T scanners: (a) ROI with the radius of 200 mm and (b) ROI with the radius of 100 mm Supporting Information File#5 The registration of the CT images with the MRI images after the software upgrade (2022) showing about 3 mm deviation on the MRI fiducial markers (left column), compared with the registration of the CT and the MRI in 2021 Supporting Information File#6 The comparison of the registration of the CT images with the MRI images taken before and after the problem mentioned in Figure 5 and Supporting Information File#5 (due to the software upgrade in 2022) was fixed: (A) before the problem fixed showing about 3 mm deviation on the MRI fiducial markers (left column), (B) after the problem fixed showing the overlap of the MRI fiducial markers with the CT fiducial markers. [file ACM2-24-e13843-s002.docx]

**Supplementary File#1**: (a) The MRID^3D^ phantom, (b) GRID^3D^ phantom, [Modus QA, Ontario Canada N6H 5L6, <https://modusqa.com/>].

**Supplementary File#2:** show the mean image distortion magnitude versus bandwidth for the ROI with the radius of (a) $200 mm$ and (b) $100 mm$ for these thirteen MRI scanners, respectively.

**Supplementary File#3**: The stability of the magnitude of MRI image distortion over 6 years of period from 2021: **(a)** ROI with the radius of $200 mm$; **(b)** ROI with the radius of $100 mm$ for GE 1.5T Artist, Siemens 1.5T Aera2, and Siemens 3T Skyra1.

**Supplementary File#4**: Comparison of the magnitude of image distortion between 1.5T and 3T scanners: (a) ROI with the radius of $200 mm$ and (b) ROI with the radius of $100 mm$.

**Supplementary File#5**: The registration of the CT images with the MRI images after the software upgrade (2022) showing about 3 mm deviation on the MRI fiducial markers (left column), compared with the registration of the CT and the MRI in 2021.

**Supplementary File#6:** The comparison of the registration of the CT images with the MRI images taken before and after the problem mentioned in **Figure 5** and **Supplementary File#5** (due to the software upgrade in 2022) was fixed: (A) before the problem fixed showing about 3 mm deviation on the MRI fiducial markers (left column), (B) after the problem fixed showing the overlap of the MRI fiducial markers with the CT fiducial markers.
