# Supplementary figures and images for: Assessment of MRI image distortion based on 6 consecutive years of annual QAs and measurements on 14 MRI scanners used for radiation therapy
Source: J Appl Clin Med Phys. 2022 Nov 16;24(1):e13843. doi: 10.1002/acm2.13843 (PMC9859981; doi:10.1002/acm2.13843)

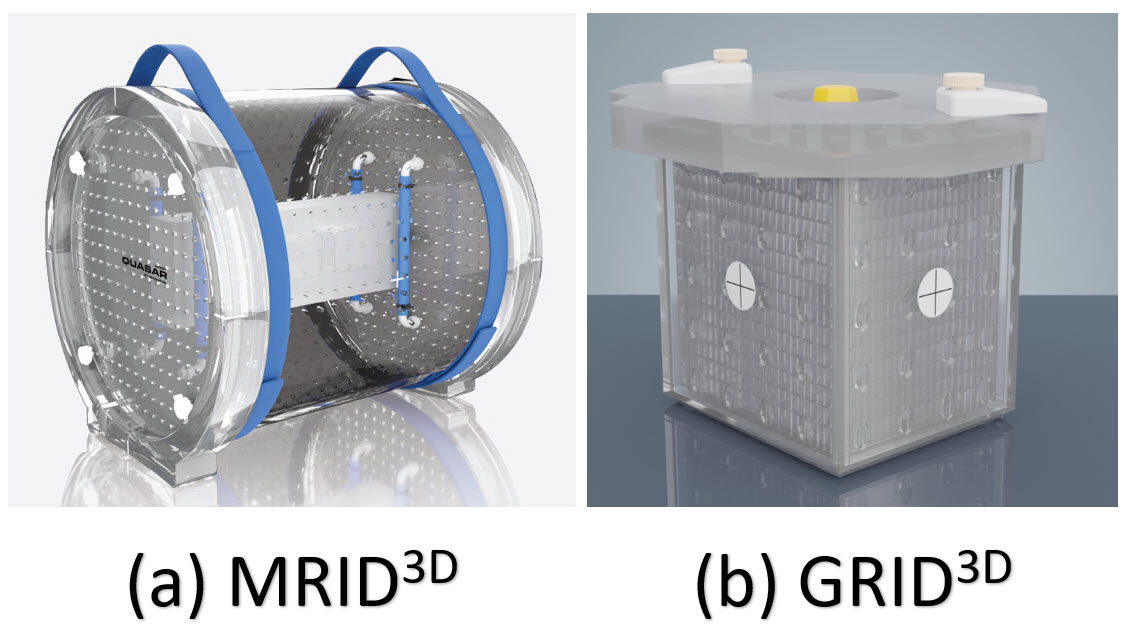

Supplement: Supplementary file 2 — Supporting Information [file ACM2-24-e13843-s001.jpg]

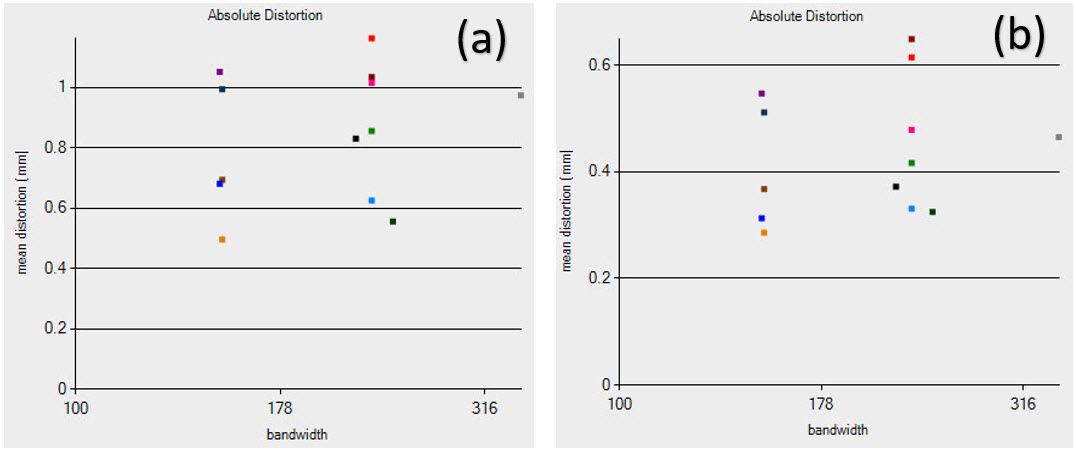

Supplement: Supplementary file 3 — Supporting Information [file ACM2-24-e13843-s005.jpg]

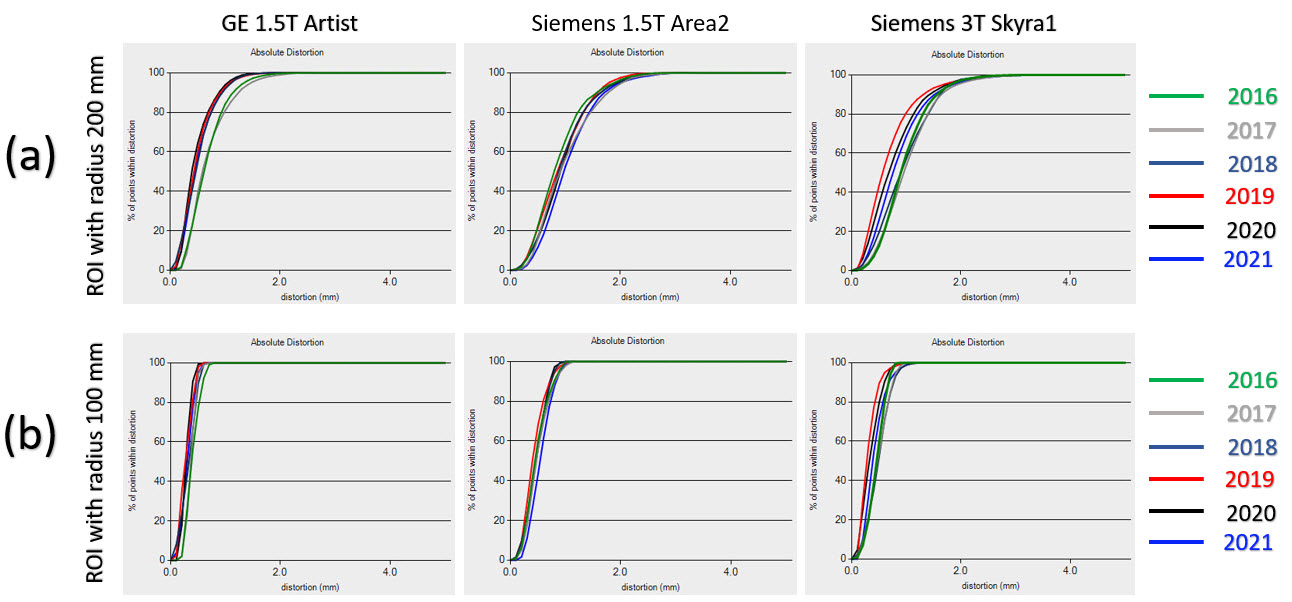

Supplement: Supplementary file 4 — Supporting Information [file ACM2-24-e13843-s004.jpg]

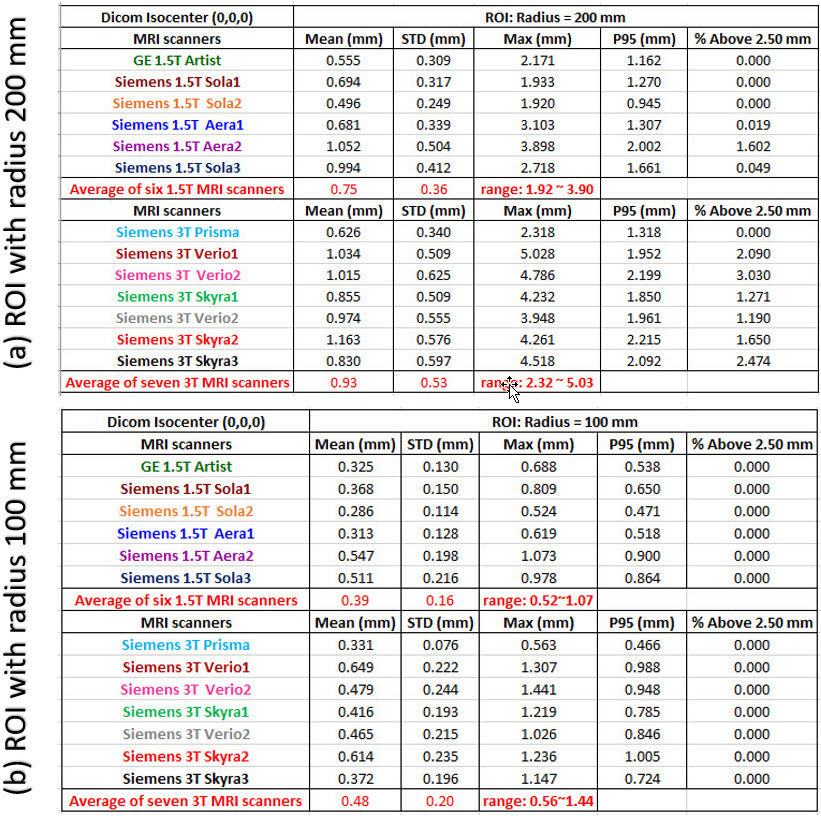

Supplement: Supplementary file 5 — Supporting Information [file ACM2-24-e13843-s003.jpg]

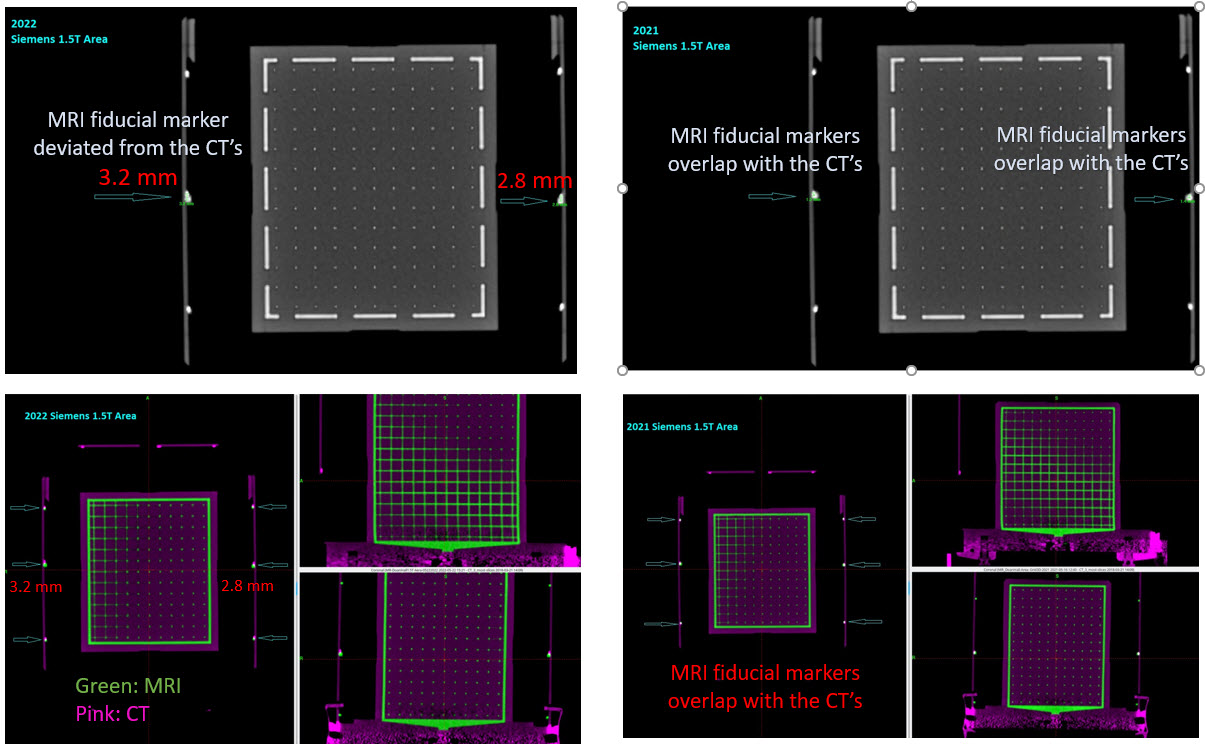

Supplement: Supplementary file 6 — Supporting Information [file ACM2-24-e13843-s007.jpg]

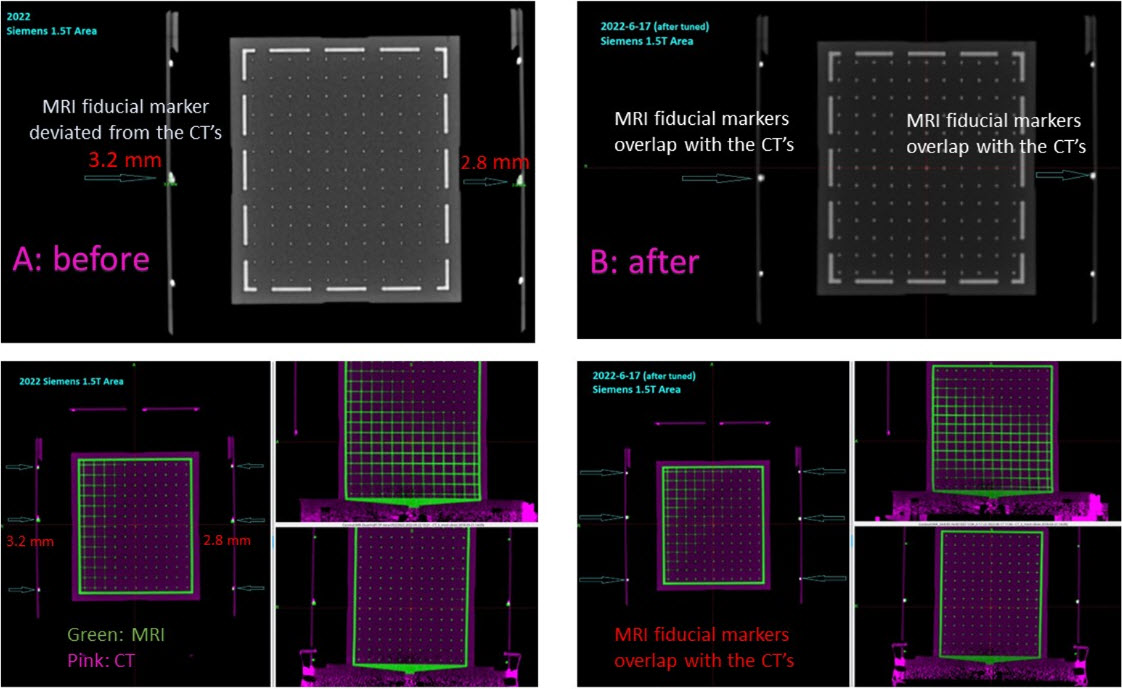

Supplement: Supplementary file 7 — Supporting Information [file ACM2-24-e13843-s006.jpg]
